# [Corrigendum] Characteristics of primary side population cervical cancer cells

**DOI:** 10.3892/ol.2025.15293

**Published:** 2025-09-23

**Authors:** Zhen-Tong Wei, Xiao-Wei Yu, Jia-Xue He, Yan Liu, Song-Ling Zhang

Oncol Lett 14: 3536–3544, 2017; DOI: 10.3892/ol.2017.6606

Following the publication of the above article, the authors have contacted the Editor to explain that, in the originally published version of Fig. 6 on p. 3543, concerning the tumorigenicity of SP and NSP cells from primary cervical cancer cells, three different injection cell quantities were applied for each type of cell. However, only two representative images were included in Fig. 6, and not the third image; the incomplete version of this figure was accidentally uploaded incorrectly owing to an oversight during the final manuscript assembly.

The revised version of Fig. 6, now showing the results of tumors developed following the injection of 1×10^5^ SP cells and NSP cells in Fig. 6C, is shown on the next page. The authors regret the error that was made in assembling this figure, and thank the Editor of *Oncology Letters* for granting them the opportunity to publish this Corrigendum. All the authors agree with the publication of this Corrigendum, and apologize to the readership for any inconvenience caused.

## Figures and Tables

**Figure 6.**
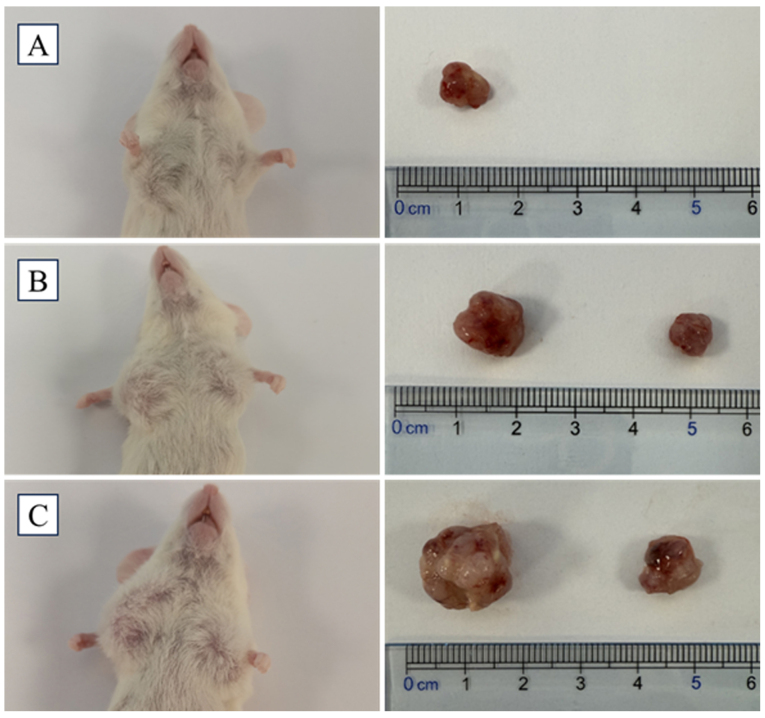
Tumorigenicity assay for SP and NSP cells isolated from patient primary cervical cancer cells. (A) Image of a mouse with a tumor developed following injection of 1×10^3^ SP cells and NSP cells. The SP cells were injected into the right forelimb, and NSP cells were injected into the left forelimbs of NOD-SCID mice. The mice were observed for 12 weeks. (B) Image of a mouse with a tumor developed following injection of 1×10^4^ SP cells and NSP cells. The SP cells were injected into the right forelimb, and NSP cells were injected into the left forelimbs of NOD-SCID mice. The mice were observed for 12 weeks. (C) Image of a mouse with tumors developed following injection of 1×10^5^ SP cells and NSP cells. Tumors developed in the right (SP cells) and left (NSP cells) forelimbs. NOD-SCID, non-obese diabetic/severe combined immunodeficiency; SP, side population; NSP, non-side population.

